# Feasibility of A Novel On-Site Detection Method for Aflatoxin in Maize Flour from Markets and Selected Households in Kampala, Uganda

**DOI:** 10.3390/toxins10080327

**Published:** 2018-08-11

**Authors:** Alex Paul Wacoo, Deborah Wendiro, Sarah Nanyonga, Joseph F. Hawumba, Wilbert Sybesma, Remco Kort

**Affiliations:** 1 Department of Molecular Cell Biology, VU University Amsterdam, De Boelelaan 1108, 1081 HZ Amsterdam, The Netherlands; wacooalex@gmail.com; 2 Yoba for Life Foundation, Hunzestraat 133-A, 1079 WB Amsterdam, The Netherlands; wilbert.sybesma@yoba4life.com; 3 Department of Nursing, Muni University, P.O. Box 725 Arua, Uganda; 4 Department of Microbiology and Biotechnology Centre, Product Development Directory, Uganda Industrial Research Institute, P.O. Box 7086 Kampala, Uganda; dwendiro@gmail.com; 5 Department of Chemistry, Faculty of Science, Kyambogo University, P.O. Box 1 Kyambogo, Uganda; snanyonga1@gmail.com; 6 Department of Biochemistry and Sports Science, School of Biological Sciences, College of Natural Sciences, Makerere University, P.O. Box 7082 Kampala, Uganda; jfh69fuuna@gmail.com; 7 TNO, Microbiology and Systems Biology, Utrechtseweg 48, 3704 HE Zeist, The Netherlands

**Keywords:** aflatoxins, maize, households, markets, immunosensor, HPLC, ELISA

## Abstract

In sub-Saharan Africa, there is a high demand for affordable and accessible methods for on-site detection of aflatoxins for appropriate food safety management. In this study, we validated an electrochemical immunosensor device by the on-site detection of 60 maize flour samples from six markets and 72 samples from households in Kampala. The immunosensor was successfully validated with a linear range from 0.7 ± 0.1 to 11 ± 0.3 µg/kg and limit of detection (LOD) of 0.7 µg/kg. The maize flour samples from the markets had a mean total aflatoxin concentration of 7.6 ± 2.3 µg/kg with approximately 20% of the samples higher than 10 µg/kg, which is the maximum acceptable level in East Africa. Further down the distribution chain, at the household level, approximately 45% of the total number contained total aflatoxin levels higher than the acceptable limit. The on-site detection method correlated well with the established laboratory-based HPLC and ELISA-detection methods for aflatoxin B_1_ with the correlation coefficients of 0.94 and 0.98, respectively. This study shows the feasibility of a novel on-site detection method and articulates the severity of aflatoxin contamination in Uganda.

## 1. Introduction

Maize is among the top ten foods consumed throughout the world. The annual production of maize is estimated to be 717 million metric tons per year globally [1]. In Africa, the highest consumption of maize has been reported to be in East and Southern Africa. Uganda is among the top 20 maize producing countries in sub-Saharan Africa, which collectively produce 96% of the total maize [2]. According to Atukwase et al. [3], maize accounts for over 40% of calories consumed per capita in both rural and urban areas of Uganda. Maize is consumed as green maize (young and soft corn prepared by either roasting or boiling immediately after harvest) and as maize flour prepared for a variety of meals, porridge or cakes [4]. A number of studies indicate that the consumption of maize contaminated with aflatoxins can be directly related to aflatoxin poisoning [4].

Aflatoxin is among the most carcinogenic toxins known to humans and is produced as a secondary metabolite by *Aspergillus flavus* and *Aspergillus parasiticus* [5]. Aflatoxin contamination in maize is attributed to the change in climatic conditions such as drought coupled with insect attacks, poor drying, and storage conditions [6,7]. Aflatoxin consumption is associated with liver cancer and immunosuppression [8]. Aflatoxins cause up to 28% of new cases of hepatocellular carcinoma worldwide every year [9]. Furthermore, aflatoxin exposure has been associated with child stunting, particularly in sub-Saharan Africa. A previous study carried out in Benin and Togo reported a 30 to 40% higher aflatoxin-albumin adduct in stunted children when compared to children with normal growth [10]. Although exposure to total aflatoxins is unavoidable, the mitigation of its risk effect is very important, thus the concentrations in food should be restricted to the lowest practical levels [11]. Regulatory limits of the concentration of aflatoxins have been globally set for food, i.e., 2 µg/kg for aflatoxin B_1_ alone and 4 µg/kg (B_1_, B_2_, G_1_, and G_2_) for the sum of aflatoxins for all cereals and all cereal products for Europe [12] and 20 µg/kg for most African countries, a limit set by the Food and Drug Administration/World Health Organization (FDA/WHO) [13]. However, the limits for total aflatoxins in milled maize in East Africa have been set at 10 μg/kg [14].

In Uganda, Kaaya and Kyamuhangire [15] found that 87% of maize kernels in humid agro-ecological zones were contaminated with mean total aflatoxin levels of 21 μg/kg. For dry and highland zones, they revealed a 78% and 69% incidence of aflatoxins contamination with a corresponding mean total aflatoxin concentration of 18 μg/kg and 12 μg/kg, respectively. In Kampala, the only reported study on aflatoxin based on five incremental samples revealed that aflatoxin contamination did not exceed 20 µg/kg in maize [16]. The exposure of household members to aflatoxins due to the consumption of contaminated maize in Kampala has not been adequately handled. The analysis of samples is performed by using high performance liquid chromatography (HPLC), enzyme linked immunosorbent assays (ELISA) and fluorescence spectrophotometers, which are laboratory based, relatively expensive, laborious and time consuming [5]. Rapid on-site detection of total aflatoxin is important for food safety management [17]. In this respect, we previously designed and constructed a simple and portable immunosensor device at the Uganda Industrial Research Institute which operates on a glass-electroless-plated Silver–Cysteine platform for the on-site detection of total aflatoxin [18]. In this study, we validated this immunosensor and evaluated maize flour in six major markets and selected households in Kampala. The results of the on-site detection of the samples were compared with those obtained by the laboratory-based techniques HPLC and ELISA.

## 2. Results

### 2.1. Validation of the Immunosensor

**Figure 1:**
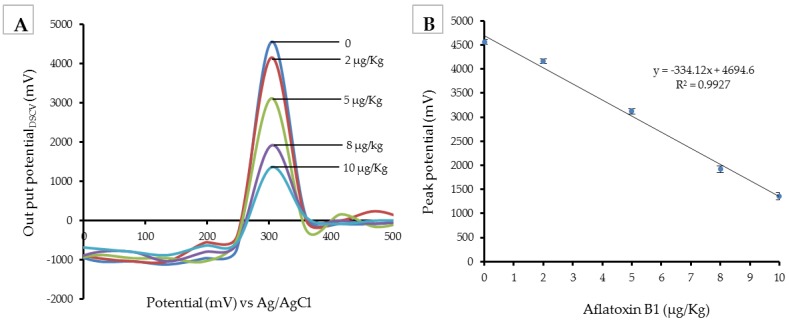
(**A**) DSCV signal recorded for HRP-blocked immune-electrode for different aflatoxin B_1_ concentrations (0–10 µg/kg) in a citrate buffer pH 7.0 (scan from 0 to 500 mV; pulse amplitude 56 mV; pulse width 460 ms, and scan rate of 20 mV/s). (**B)** A calibration curve of peak DSCV potential (mV) versus aflatoxin B_1_ concentration (µg/kg).

The performance of the electrochemical immunosensor for the analysis of aflatoxin B_1_, operating on the electroless-plated Silver/Cysteine sensor platform, was validated as described in Section 4.2. The differential staircase voltammogram signal and the standard curve generated from the sensor are shown in Figure 1. The limit of detection (LOD), linear range, precision, and accuracy are shown in Table 1. The limit of detection (LOD), defined as the lowest amount of aflatoxin B_1_ that can be detected, was found to be 0.7 µg/kg. The sensor could detect concentrations up to 11 µg/kg, implying that it operated well in the linear range from 0.7 ± 0.1 to 11 ± 0.3 µg/kg. Additionally notable was the fact that within the linear range, the differential staircase voltammogram peak heights increased exponentially with a decrease in aflatoxin B_1_, indicating that within the linear range (0.7 ± 0.1 to 11 ± 0.3 µg/kg), the biosensor could be used reliably.

The precision of the immunosensor determined as the coefficient of variation between the triplicate results of each spiked aflatoxin B_1_ (2, 5 and 10 µg/kg) concentration is shown in Table 1. The immunosensor exhibited very high precision as demonstrated by the very low coefficient of variations (CVs) (Table 1) of 0.3% and 1.5% for intra-day and inter-day, respectively. The immunosensor showed good recovery values of 99.0 ± 1.5%, 88.2 ± 0.8%, and 70.5 ± 0.3% corresponding to spiked values of 2, 5, and 10 µg/kg, respectively.

### 2.2. On-Site Detection

#### 2.2.1. Market Samples

**Figure 2:**
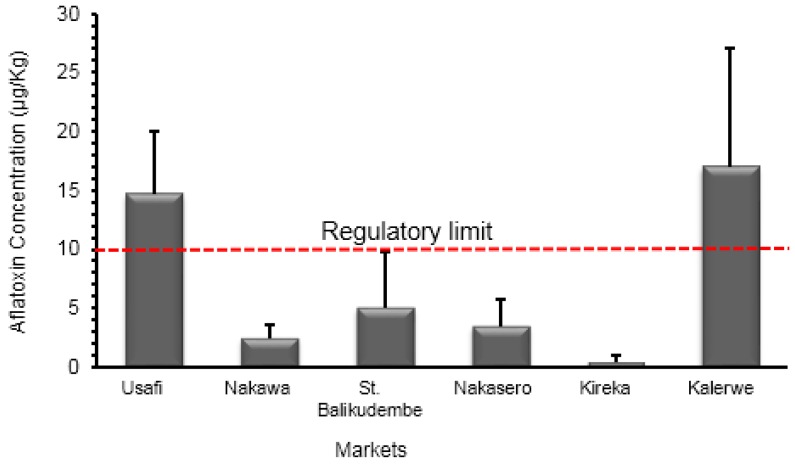
Total aflatoxin levels in maize flour for human consumption in the six major markets of Kampala and the regulatory limit for the East Africa Community [14].

The levels of total aflatoxin contamination of maize in the markets of Usafi, Nakawa, St. Balikudembe, Nakasero, Kireka, and Kalerwe are shown in Figure 2. The market samples had a mean aflatoxin concentration of 7.6 ± 2.3 µg/kg. From the total 60 samples analyzed, 35% (21/60) contained detectable levels of aflatoxins with the concentration ranging within the limit of quantification of 0.7 µg/kg to 88.6 µg/kg.

A total of 20% (12/60) of the maize flour samples with a mean total aflatoxin concentration of 32.7 ± 6.3 µg/kg exceeded the East African regulatory limit of 10 μg/kg [14]. The Kalerwe samples registered the highest mean total aflatoxin level of 17 ± 10.1 µg/kg with 30% (3/10) of the samples above the permissible limit of 10 µg/kg. For instance, one of the samples contained the highest total aflatoxin concentration of 88.6 µg/kg, which is approximately eight fold of the East Africa regulatory limit of 10 µg/kg. Usafi emerged second after the Kalerwe market with a mean total aflatoxin level of 14.7 ± 5.3 µg/kg and 50% (5/10) of samples had a total aflatoxin concentration higher than the East Africa regulatory set limit of 10 µg/kg. The Nakasero market contained two samples with total aflatoxin levels above 10 µg/kg. The St. Balikudembe and Nakawa markets each contained one sample above the limit with 51.9 µg/kg and 12.9 µg/kg, respectively, while all Kireka samples had a total aflatoxin concentration below the East Africa regulatory limit.

**Figure 3:**
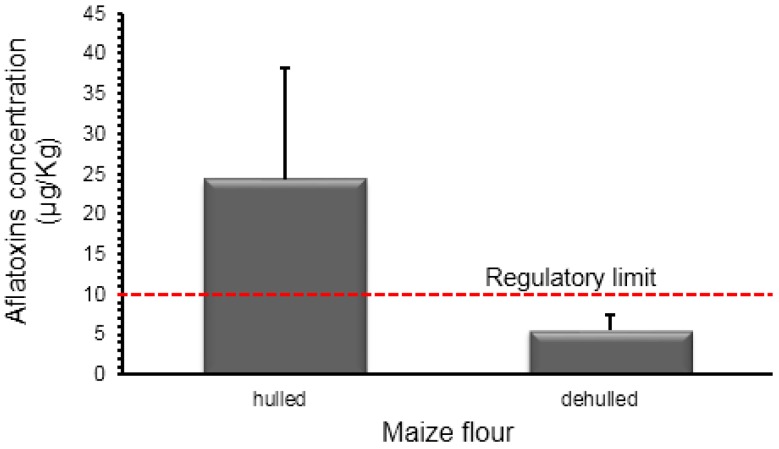
Total aflatoxin levels in hulled and dehulled maize flour and the regulatory limit for the East African Community.

The levels of total aflatoxin concentration in both dehulled and hulled maize flour samples are shown in Figure 3. Approximately 83.3% of the hulled maize samples were detected as positive for aflatoxins, with the mean total aflatoxin concentration of 24.2 ± 13.9 µg/kg. The mean total aflatoxin level of the hulled maize samples was above the East African regulatory limit of 10 µg/kg. Approximately 50% of the hulled maize samples contained detectable aflatoxin levels above the East Africa regulatory limit. In comparison, less than 15% of the dehulled maize samples contained a total aflatoxin concentration above the East Africa regulatory limit. The mean total aflatoxin concentration of the dehulled maize was 5.6 ± 1.8 µg/kg.

#### 2.2.2. Households

**Figure 4:**
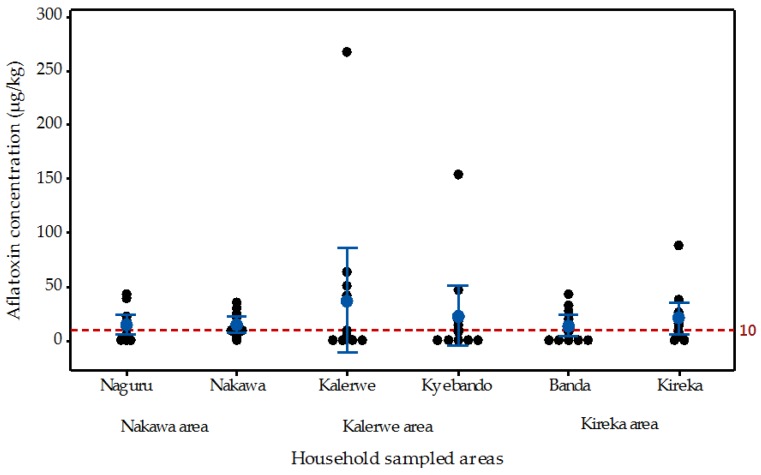
Individual value plot of total aflatoxin concentration of maize flour samples from some selected Kampala households and the regulatory limit for the East African Community.

The levels of total aflatoxin contamination of the maize samples collected from randomly selected households in Kampala are shown in Figure 4. Approximately 74% (53/72) of the household samples tested positive for aflatoxins. The household samples contained a mean total aflatoxin concentration of 22.2 ± 4.6 µg/kg, which was two times higher than the regulatory limit of 10 µg/kg. Furthermore, out of the 53 samples with detectable concentrations of aflatoxin, approximately 65% contained concentrations above the set East Africa regulatory limit of 10 µg/kg. One sample was found with a total aflatoxin concentration of 268 µg/kg. Such a concentration is more than 26 fold higher than the East Africa regulatory limit.

Generally, the mean concentration of total aflatoxins in the households from all sampled areas were higher than the 10 µg/kg minimum regulatory level set by the East Africa community. Areas close to the Nakawa market (Naguru and Nakawa) had mean total aflatoxin concentrations of approximately 15 µg/kg. Consumers eating unhulled maize flour from Kalerwe are at very high risk. Maize flour sampled from households in Kalerwe contained a mean total aflatoxin concentration of approximately 35 µg/kg and those in Kyebando were approximately 23 µg/kg. Maize flour sampled from households in the Kireka and Banda area showed mean total aflatoxin levels of approximately 20 µg/kg and 14 µg/kg, respectively.

#### 2.2.3. Comparison between Aflatoxin Contamination of Households and Market Samples

**Figure 5:**
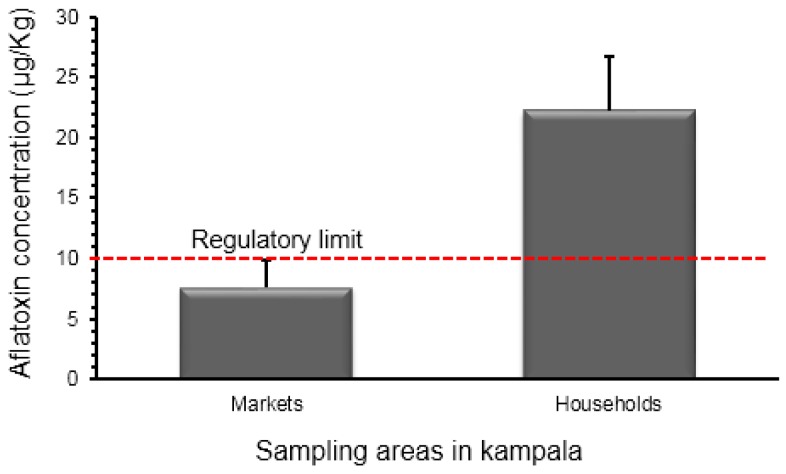
Comparison between markets and household total aflatoxin contamination levels and the regulatory limit for the East Africa Community.

The concentrations of total aflatoxin from the samples collected from the market and households in Kampala are shown in Figure 5. The mean total aflatoxin concentration (7.6 ± 2.3 µg/kg) of the market samples was approximately a factor of three times less than the 22.2 ± 4.6 µg/kg from the households. Only 20% of the samples from the markets were above the set regulatory limit of 10 µg/kg. In comparison, nearly 50% of the households had a considerably high and unacceptable level of aflatoxins above the East Africa regulatory limit of 10 µg/kg. Moreover, the highest household aflatoxin concentration level was found to be as high as 268 µg/kg, which was approximately three times higher (88.6 µg/kg) than that found at the markets.

### 2.3. On-Site Detection

**Figure 6:**
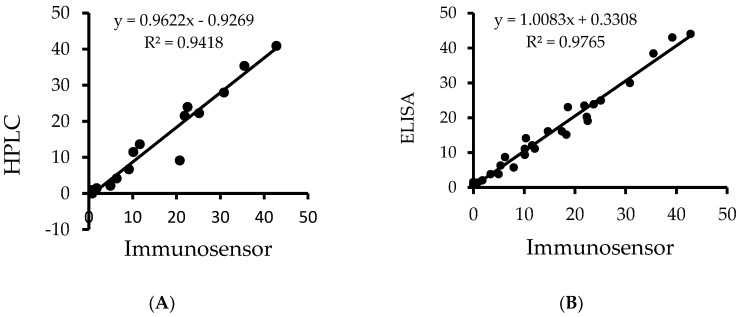
Regression curves for aflatoxin B_1_ (μg/kg) in maize flour: (**A**) the novel immunosensor versus HPLC-fluorescence. (**B**) the novel immunosensor versus ELISA.

The correlation curve of aflatoxin B_1_ in corn flour analyzed using the immunosensor and the HPLC from the same sets of samples is shown in Figure 6A. A correlation coefficient (R^2^) of 0.94 was obtained with linear regression equation y = 0.88x + 0.53 for the 15 samples of corn flour analyzed.

The correlation curve developed from the analysis by the immunosensor and ELISA is shown in Figure 6B. The correlation coefficient of 0.98 and a linear regression equation of y = 1.01x + 0.33 were obtained for 30 samples.

## 3. Discussion

The contamination of maize with aflatoxins is an ongoing public health problem for communities that produce, trade, and consume the product [19,20]. The control of aflatoxin contamination of food requires sensitive analytical methods such as HPLC and ELISA. However, these methods are only limited for use in the laboratory set up [21,22]. Thus, we developed a novel immunosensor method for the on-site detection and quantification of total aflatoxins (Figure S1) [18].

The applicability of the novel immunosensor in estimating the concentration of aflatoxins in maize flour samples was confirmed and shown to be a suitable on-site means of aflatoxin determination, with excellent recovery values ranging from 70.5 ± 0.3% to 99 ± 1.5%. The accuracy was well within the recommended recovery values of 70–110% for aflatoxin concentrations up to 10 µg/kg set by the European Commission (EC) [23,24]. The very low coefficient of variations (CVs) exhibited by the immunosensor of 0.3% and 1.5% for the intra-day and inter-day measurements, respectively, met the recommendations for immunosensor validation [25]. Furthermore, the immunosensor demonstrated an excellent correlation coefficient (R^2^ = 0.94 and 0.98) when the results were compared to those obtained with HPLC and ELISA. The obtained values were comparable to those previously reported in [26], implying that the novel immunosensor was suitable for the analysis of aflatoxin B_1_ concentration in the maize samples. However, the polyclonal anti-aflatoxin B_1_ antibody used was not only reactive to aflatoxin B_1_, but also to B_2_ and G_2_ [27]. Despite differences in extraction method, the anti-aflatoxin antibody used and the detection method, there was no statistically significant difference between the values obtained for total aflatoxins by the novel immunosensor, and the HPLC or ELISA methods used aflatoxin levels in naturally contaminated maize samples.

The situation of total aflatoxin contamination of maize is alarming, particularly in East Africa where maize forms approximately 40% of the daily diet. In contrast to a previous study [16], approximately 35% of the market samples tested positive for aflatoxins and nearly 60% of the positive samples exceeded the East Africa set regulatory limit of 10 µg/kg. Similarly, Perrone et al. [28] found that 30% of 56 maize samples collected from markets in Nigeria and Ghana were contaminated with total aflatoxin in the concentration range from 0.5 to 480 µg/kg. Probst et al. [29] revealed a total aflatoxin level of up to 435 µg/kg in maize sampled from farmers’ fields or small local markets. The aflatoxin contamination of maize collected from the markets could be attributed to the inability of both farmers and traders to demonstrate compliance with high quality maize processing and storage at different stages in the supply chain [30]. The traders demonstrate the quality of their maize flour through touching, biting, and tasting (Figure S2A,B), which cannot detect contaminations with aflatoxin in particular. The available analytical methods (HPLC, ELISA, fluorescence spectrophotometer) are not suited for the analysis of samples in the field [5] and are located in the central laboratories far away from stores with the bulk product. Moreover, the time lag between sending the samples from the farmers to the laboratory up to when the traders can access their results is approximately two weeks [31]. In addition, the analytical services are very expensive, costing more than $30 USD. Hence, for a typical farmer producing on average less than 100 kg (costing approximately $22.22 USD) of maize per season, testing for aflatoxin contamination is neither feasible nor practical.

The consumer preference between the hulled and dehulled maize has been reported in East Africa [32]. From that study, approximately 50% of the consumers ate hulled or a mixture of hulled and dehulled maize. The current study, however, revealed that consuming hulled maize predisposed consumers to a higher risk of aflatoxin poisoning. The mean total aflatoxin concentration in hulled maize was found to be very high (24.2 ± 13.9 µg/kg), approximately four times that of dehulled maize (5.6 ± 1.8 µg/kg). Dehulling was reported to have reduced 92% of the aflatoxin concentration levels in maize meals [33]. The process of dehulling maize removes the husk which contains the vitamins and mineral salts and therefore makes the maize less vulnerable to fungal attack and aflatoxin contamination [34,35]. Though hulled maize has a higher nutritional value, it is not preferred by consumers due to its color and poor organoleptic properties. However, in some regions of East Africa, the availability and consumption of hulled maize is common due to the introduction of low-cost, small hammer mills, which lack the provision for dehulling [4].

In the current study, very high total aflatoxin contamination levels of up to 74% of the household samples were revealed. Nearly 50% of these positive samples contained aflatoxin with concentrations between 10 µg/kg and 268 µg/kg, which were similar to those reported in a previous study in Kenya [36]. In addition, the number of positive samples and the overall total aflatoxin concentration levels in the household samples was three to four times higher than those in the samples obtained from the market. It is very important to note that most markets in Kampala act as a wholesale point for retail shops and household consumers. It could be anticipated that the household consumers acquire the maize in bulk of either 25 or 50 kg and consume this for the next few weeks or buy from the nearby retail shops. The relatively long storage time of maize flour in retail shops or households may explain the high contamination levels of maize in the households.

Our recently developed on-site immunosensor was successfully validated and did not produce any significant differences in results obtained by either HPLC or ELISA. Our study therefore demonstrated the feasibility of a novel on-site method in assessing total aflatoxin concentration levels, which is a very important step towards the proper management of aflatoxins in maize grain. This study also revealed that down the distribution chain of maize flour, there was an increase in the contamination levels of aflatoxin. Thus, household maize consumers are at a very high risk of aflatoxin poisoning and therefore it is highly recommended that methods such as the proper drying of grains, and proper storage conditions should become common practice to minimize exposure to aflatoxin.

## 4. Materials and Methods

### 4.1. Sample Collection

Kampala is the capital and largest city of Uganda and has been divided into five divisions (Nakawa, Kawempe, Rubaga, Makindye, and Kampala Central). The maize flour samples used in this study were collected from six markets (Kalerwe, Usafi, Kireka, St. Balikudembe, Nakasero, and Nakawa) that are considered to have the highest number of stalls. Three markets (St. Balikudembe, Nakasero, and Usafi) were from Kampala central, two (Kireka and Nakawa) from Nakawa, and one (Kalerwe) from Kawempe. In every market, ten samples were taken from ten different bags containing 50–100 kg of maize flour resulting in a total of 60 samples. The bags were selected randomly from 10 different stalls and the samples were taken by purchasing four times 250 g from the top to the bottom of each bag using a locally made spear-like spoon. Next, all four samples of 250 g of maize were pooled and blended thoroughly [37]. Out of the ten samples from each market, five were dehulled and five hulled. For the markets that did not have hulled maize for sale, the dehulled maize flour was taken as a substitute.

The household samples were taken randomly from Kireka, Banda, Nakawa, Kalerwe, and Kyebando, which are close to the Kalerwe, Nakawa and Kireka markets. The St. Balikudembe, Nakasero, and Usafi markets are located in the center of Kampala city with no nearby households. An approximately 50 g sample of maize flour was taken from each household. At least 12 samples were taken from each selected area, leading to a total of 72 samples.

### 4.2. Validation of the Novel Immunosensor

The validation of the immunosensor was done according to the procedure described by the International Conference on Harmonization (ICH) (1995) [38]. The limit of detection (LOD) and linear range were determined by using concentrations of aflatoxin B_1_ standard (0, 0.2, 0.5, 0.8 and 1.0 ng/mL) prepared in 10% (*v/v*) methanol as described by Wacoo et al. [18]. In order to detect the total aflatoxins with the immunosensor, we used the anti-aflatoxin B_1_ polyclonal antibody (Merck, Dorset, England, UK) produced in rabbit. Accordingly, the ‘total aflatoxin’ detected by the immunosensor used in this study was defined as the sum of aflatoxins reacting with the anti-aflatoxin B_1_ polyclonal antibody, which included aflatoxin B_1_, B_2_, and G_1_, but not B_2a_, G_2_, G_2a_, or M_1_ [27]. In order to evaluate the precision and accuracy of the immunosensor, the blank maize flour (Maganzu Millers, Kampala, Uganda) was first screened for aflatoxin B_1_ contamination using thin layer chromatography (TLC) [39]; to ascertain that the maize flour samples were free of aflatoxins, 0.5 g was spiked with 125 µL of 0.2 ng/mL of aflatoxin B_1_. The same procedure was then subsequently repeated with 0.5 and 1.0 ng/mL concentrations of aflatoxin B_1_. The precision was expressed as the degree of scatter (coefficient of variation) between three measurements of aflatoxin B_1_ from spiked maize samples taken every two hours for a total of six hours. The precision was determined for inter-day and intraday. The accuracy was estimated as the recovery at three different aflatoxin concentration levels (0.2, 0.5 and 1.0 ng/mL) [38].

### 4.3. On-Site Detection

Aflatoxins in the various maize samples were extracted by suspending two grams of maize flour in 10 mL of 70% (*v/v*) methanol. The suspensions were subsequently homogenized by shaking for approximately five minutes at room temperature. One hundred microliter (100 µL) aliquot from each suspension was diluted with 600 µL of distilled water to reduce the concentration of methanol to 10% (*v/v*). Subsequently, the aflatoxin levels were measured by the use of a novel immunosensor [18].

### 4.4. Laboratory Control

#### 4.4.1. HPLC

Fifteen maize flour samples that were analyzed by using the immunosensor were randomly taken for the detection of aflatoxin levels by HPLC (Shimadzu, Tokyo, Japan) as described by Muscarella et al. [40] with modifications from the Uganda Bureau of Standards (UNBS). The total aflatoxins from the maize flour samples were extracted by suspending 15 g into 30 mL of 80% (*v/v*) methanol and vortexed for approximately 3 minutes and subsequently filtered (Whatman filter no. 40). An aliquot of 2 mL of each extract was mixed with 8 mL of phosphate buffered saline (PBS) prior to cleaning through the immunoaffinity column (Aflastar R IAC, Romer lab, Getzersdorf, Austria) previously equilibrated with 10 mL of PBS, pH 7.4, at a flow rate of 0.5 mL/min. The Aflastar immunoaffinity columns contain monoclonal antibodies that react with aflatoxin B_1_, B_2_, G_1_, and G_2_. The column was then washed with 4 mL of distilled water to remove any unbound molecules and the bound aflatoxins were eluted using 2 mL methanol followed by 1 mL HPLC grade water. Aliquots of 20 µL each of the extract were injected into the HPLC column previously equilibrated with methanol, and the aflatoxins eluted using methanol:acetonitrile:water (8:27:65, *v/v*) mobile phase, at a flow rate of 0.7 mL/min. Detection was performed by a fluorescence detector operated at excitation and emission wavelengths set at 365 and 450 nm, respectively. Accordingly, the results used per sample was the sum of the detected aflatoxins (total aflatoxins). Correlation analysis was performed between the results of the two methods. The correlation curve was then obtained by plotting the results obtained by HPLC against the immunosensor results for the same sets of samples.

#### 4.4.2. ELISA

Thirty maize flour samples analyzed by immunosensor were randomly taken for the detection of aflatoxin concentrations using the ELISA kit Ridascreen^®^ Aflatoxin Total (R-Biopharm, Darmstadt, Germany), a competitive enzyme immunoassay with mono-clonal anti-aflatoxin antibodies and cross-reactivity of 100% with aflatoxin B_1_, 48% with B_2_, 75% with G_1_, and 18% with G_2_ [41]. Briefly, 2 g of maize flour was extracted with 10 mL of 70% (*v/v*) methanol. The suspensions were subsequently mixed using a VWR ADC 3500 Shaker (BioSurplus, Inc, San Diego, CA, USA) for 10 min at room temperature. The samples were then centrifuged for 10 min at a centrifugal force of 3500 g at room temperature and the supernatants were collected. Approximately 50 μL of each supernatant was analyzed using the ELISA. The results were used for comparative analysis with the results of the immunosensor in a correlation curve.

## Figures and Tables

**Table 1:** Validation parameters of the immunosensor.

Parameters	Values
Limit of Detection (LOD) (µg/kg)	0.7
Linear range (µg/kg)	0.7 ± 0.1 to 11 ± 0.3
Precision (CV) (%)	0.3 (intra-day)
Accuracy	1.5 (inter-day)
aflatoxin B_1_ standard(µg/kg)	2
recovery (%)	99.0 ± 1.5
aflatoxin B_1_ standard(µg/kg)	5
recovery (%)	88.2 ± 0.8
aflatoxin B_1_ standard(µg/kg)	10
recovery (%)	70.5 ± 0.3

## Supplementary Materials

The following are available online at http://www.mdpi.com/2072-6651/10/8/327/s1, Figure S1: The image of the assembled portable electrochemical immunosensor designed for on-site detection of aflatoxins, Figure S2: Current practice of ‘quality and safety assessment’ of flour at the Bweyale Market in the Kirandongo District, Uganda. (A) Evaluation of texture by the customer, and (B) Evaluation of taste (bitterness) by the saleswoman.
