# Supplementary material for: Feasibility of A Novel On-Site Detection Method for Aflatoxin in Maize Flour from Markets and Selected Households in Kampala, Uganda
Source: Toxins (Basel). 2018 Aug 11;10(8):327. doi: 10.3390/toxins10080327 (PMC6115733; doi:10.3390/toxins10080327)
Supplement: Supplementary file 1 [file toxins-10-00327-s001.pdf]

Supplementary Materials: Feasibility of A Novel On-Site Detection Method for Aflatoxin in Maize Flour from Markets and Selected Households in Kampala, Uganda

Alex Paul Wacoo, Deborah Wendiro, Sarah Nanyonga, Joseph F. Hawumba, Wilbert Sybesma and Remco Kort

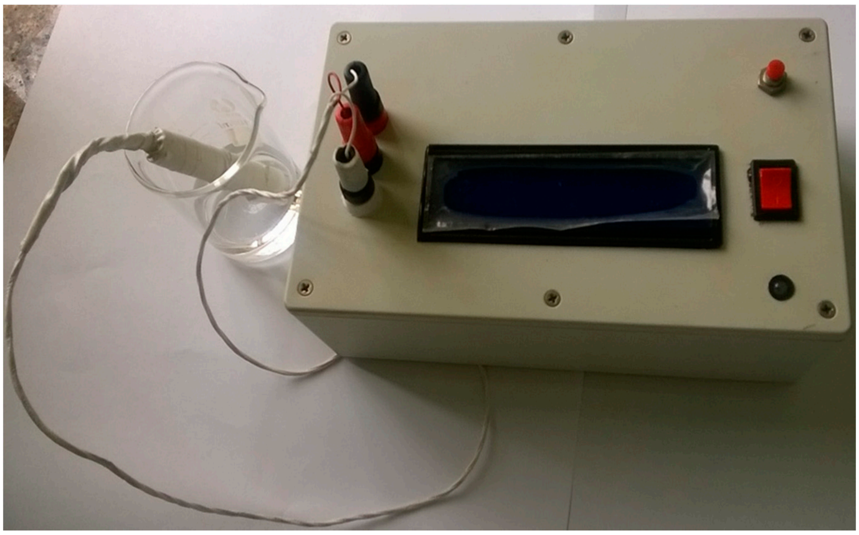

Figure S1. The image of the assembled portable electrochemical immunosensor designed for the on-site detection of aflatoxins.

The operation of the immunosensor has been previously reported by Wacoo et al. [1]. Data acquisition, analysis and display were performed by a locally designed potentiostat enclosed in a small casing. The device is portable, weighs approximately 450 g and 19 cm × 11 cm × 6 cm in size and is powered by replaceable batteries and therefore well-suited for on-site application [2].

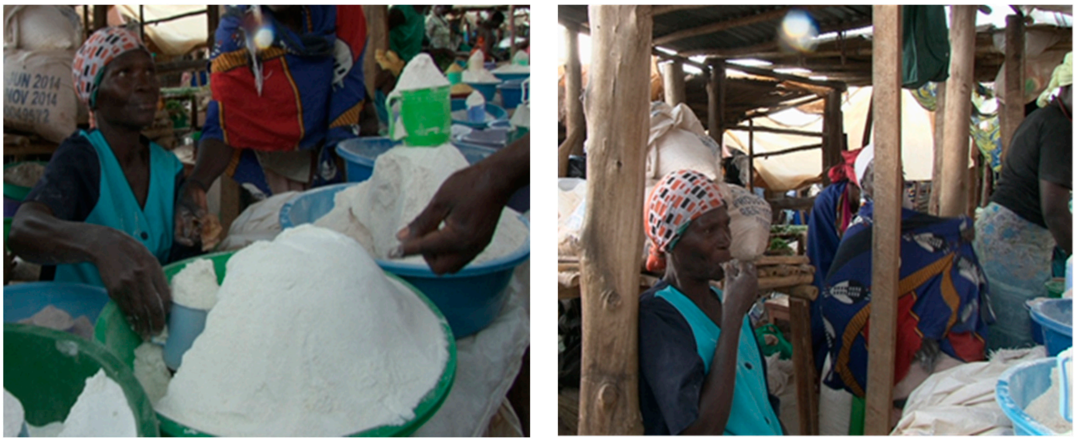

(**A**) (**B**)

Figure S2. Current practice of 'quality and safety assessment' of flour at the Bweyale Market in the Kirandongo District, Uganda. (**A**) Evaluation of texture by the customer, and (**B**) Evaluation of taste (bitterness) by the saleswoman.

References

1. Wacoo, P.A.; Ocheng, M.; Wendiro, D.; Vuzi, P.C.; Hawumba, F.J. Development and characterization of an electroless plated silver/cysteine sensor platform for the electrochemical determination of aflatoxin B_1. *J. Sensors* **2015**, *2016*, 1–8.
2. Ocheng M, W.P.A., Wendiro D., Vuzi P. C and Hawumba F. J. The assembly of a field-deployable biosensor device operating on the electroless silver plated sensor platform for detection of aflatoxin B_1. *Int. J. Sci. Eng. Investig.* **2015**, *4*, 6–10.
